# A Comparative Analysis of the Novel Conditional Deep Convolutional Neural Network Model, Using Conditional Deep Convolutional Generative Adversarial Network-Generated Synthetic and Augmented Brain Tumor Datasets for Image Classification

**DOI:** 10.3390/brainsci14060559

**Published:** 2024-05-30

**Authors:** Efe Precious Onakpojeruo, Mubarak Taiwo Mustapha, Dilber Uzun Ozsahin, Ilker Ozsahin

**Affiliations:** 1 Department of Medical Diagnostic Imaging, College of Health Sciences, University of Sharjah, Sharjah 27272, United Arab Emirates; dozsahin@sharjah.ac.ae; 2 Research Institute of Medical and Health Sciences, University of Sharjah, Sharjah 27272, United Arab Emirates; 3 Operational Research Centre in Healthcare, Near East University, TRNC Mersin 10, Nicosia 99138, Turkey; efeprecious.onakpojeruo@neu.edu.tr (E.P.O.);; 4 Department of Biomedical Engineering, Near East University, TRNC Mersin 10, Nicosia 99138, Turkey; 5 Brain Health Imaging Institute, Department of Radiology, Weill Cornell Medicine, New York, NY 10065, USA

**Keywords:** augmented dataset, brain tumor, C-DCNN model, DCGAN, GANs, image datasets, kidney tumor, synthetic dataset, model performance

## Abstract

Disease prediction is greatly challenged by the scarcity of datasets and privacy concerns associated with real medical data. An approach that stands out to circumvent this hurdle is the use of synthetic data generated using Generative Adversarial Networks (GANs). GANs can increase data volume while generating synthetic datasets that have no direct link to personal information. This study pioneers the use of GANs to create synthetic datasets and datasets augmented using traditional augmentation techniques for our binary classification task. The primary aim of this research was to evaluate the performance of our novel Conditional Deep Convolutional Neural Network (C-DCNN) model in classifying brain tumors by leveraging these augmented and synthetic datasets. We utilized advanced GAN models, including Conditional Deep Convolutional Generative Adversarial Network (DCGAN), to produce synthetic data that retained essential characteristics of the original datasets while ensuring privacy protection. Our C-DCNN model was trained on both augmented and synthetic datasets, and its performance was benchmarked against state-of-the-art models such as ResNet50, VGG16, VGG19, and InceptionV3. The evaluation metrics demonstrated that our C-DCNN model achieved accuracy, precision, recall, and F1 scores of 99% on both synthetic and augmented images, outperforming the comparative models. The findings of this study highlight the potential of using GAN-generated synthetic data in enhancing the training of machine learning models for medical image classification, particularly in scenarios with limited data available. This approach not only improves model accuracy but also addresses privacy concerns, making it a viable solution for real-world clinical applications in disease prediction and diagnosis.

## 1. Introduction

Medical researchers and practitioners play a critical role in disease prediction, diagnosis, and treatment. However, a significant challenge faced by these professionals is the limited availability of adequate and diverse datasets [1,2]. The success of predictive models in medical disease analysis heavily relies on the quantity of data used for training [3,4]. To address these limitations, researchers have turned to augmentation techniques and the creation of synthetic datasets as practical solutions to expand and enhance the available data.

Some experts and researchers, for example [5], and many more, often mistake image augmentation to be the same thing as synthetic datasets. However, there is a clear difference between augmentation and synthetic datasets. Image augmentation involves manipulating the training set by changing its geometric and color space properties (such as rotation, scaling, cropping, brightness, zooming, and contrast) [6]. One major disadvantage of using primary data or augmented data is that there is always a risk of an accidental breach and lack of preservation of the privacy of patients represented by the data, as data collection through Magnetic Resonance Imaging (MRI) or Computed Tomography (CT) scans could contain images of the head, facial images, or comparable representations in a manner that allow the identities of research participants to be readily and accurately ascertained [7,8]. On the other hand, synthetic data can be used to circumvent this hurdle while also enhancing the performance of models where data size is not adequate to train models. Hence, synthetic datasets refer to artificially generated data that closely resemble the characteristics of the primary datasets. The original dataset is not utilized in the creation of synthetic data; rather, it is generated artificially to produce new sets of datasets that serve as supplementary or alternative sources of data, particularly in scenarios where there is a dearth of sufficient authentic data. The generation of synthetic datasets typically entails the utilization of generative models, such as Generative Adversarial Networks (GANs), Variational Autoencoders (VAEs), or other methodologies, such as rule-based generators or simulation models [9].

The utilization of GANs for generating synthetic data in medical research is a relatively novel approach that has not been extensively explored in the existing literature. For example, in the context of brain tumor prediction, GANs have not been widely studied for generating synthetic datasets. While there have been some applications of GANs in medical image synthesis for brain tumor segmentation in studies by Myronenko et al., Huang et al., and Cirillo et al. [10,11,12], the use of GANs, specifically for generating synthetic data to improve brain cancer prediction models, remains relatively unexplored. In cardiac disease diagnosis, the availability of large and diverse datasets is crucial for training accurate predictive models [13]. While augmentation techniques have been explored in this domain, typically in a study by Anwar et al. [14], the use of GANs to generate synthetic data for improving cardiac disease diagnosis models remains underexplored. The use of GANs for generating synthetic medical images to augment existing datasets is an emerging field of research (e.g., for retinal image synthesis in diabetic retinopathy diagnosis) [15]. However, a comprehensive investigation comparing the impact of GAN-generated synthetic images versus traditional augmentation techniques on the performance of medical image analysis models is relatively limited. While GANs have shown promise in generating synthetic data for various applications, their application, specifically for disease prediction and diagnosis, remains a relatively unexplored territory in the existing literature. This study’s focus on using GANs to create synthetic datasets and directly comparing their performance with classical augmentation techniques in the context of disease prediction, particularly for brain tumors, contributes to filling this gap and adding valuable insights to the field of medical research. 

This study seeks to make several significant contributions to the domain of disease prediction, specifically for brain tumors. Because of the stressful lifestyle of humans all over the world, brain tumor occurrences are on a steady rise at an alarming rate. If a brain tumor is not detected at an early stage, it can have deleterious consequences on mortality. The first contribution of this study is to develop a novel Conditional Deep Convolutional Neural Network (C-DCNN) model from CNN architectures that are capable of detecting tumors and differentiating brain tumors from kidney tumors and then conduct a thorough comparison of the performance of our novel C-DCNN model with state-of-the-art models such as ResNet50, VGG19, InceptionV3, and VGG16 when trained on augmented datasets versus synthetic datasets. By analyzing the effectiveness of these two approaches, the study offers insights into which method leads to better predictive performance. Secondly, the research focuses on assessing the impact of using synthetic datasets on the prediction accuracy of disease models, specifically for brain tumors and kidney tumors. Understanding how synthetic datasets affect the performance of predictive models is essential for developing more accurate and reliable diagnostic tools for medical professionals. Moreover, this study undertakes a comprehensive evaluation of the potential advantages and limitations of utilizing synthetic datasets compared to augmented datasets in disease prediction. Identifying the strengths and weaknesses of each approach can guide researchers and practitioners in making informed decisions regarding dataset selection for their specific medical studies.

## 2. Related Studies

The concept of GANs was initially presented by Goodfellow and colleagues in their study [16]. The researchers employed the backpropagation approach to train their multi-layer perceptron models for generators and discriminators. Over the years, GANs have gained a lot of popularity as a result of numerous modifications that have improved the quality of the images that are created and expanded the range of applications that may be used with them. A specific architectural design known as the Deep Convolutional Generative Adversarial Network (DCGAN), according to Radford et al. [17], aims to lessen the distinction between Convolutional Neural Networks (CNNs) utilized for supervised and unsupervised learning. Using the least squares loss function for the discriminator, as stated by Mao et al. [18], the Least Squares Generative Adversarial Network (LSGAN) is able to increase both the stability of training and the quality of the images that are produced. Study [19] proposed the use of a Conditional Generative Adversarial Network, also known as a CGAN. The discriminator and generator in this network were taught to generate images by integrating class labels. This was accomplished through training [20]. Through the process of combining the Deep Convolutional Generative Adversarial Network (DCGAN) and the Conditional Generative Adversarial Network (CGAN), the Conditional Deep Convolutional Generative Adversarial Network (C-DCGAN) was created. Feature extraction from the C-DCGAN model was accomplished by Luo et al. [21] through the utilization of a convolutional neural network. There is also a conditional extension included to enhance the data. Another model called the InfoGAN model was introduced by Chen et al. [22] and is built on an information-theoretic framework, allowing for the manipulation of the latent space. This model makes it possible to manipulate the latent space. In addition, the sleep–wake algorithm is incorporated into the process of training InfoGAN [23]. Similarly, the Wasserstein Generative Adversarial Network (WGAN), proposed by [24], has been developed as an improved training technique to solve the issues associated with mode collapse and to provide improved learning curves. As stated by Dharanya et al. [25], label conditioning is an essential element that is included in the Auxiliary Classifier Generative Adversarial Network (ACGAN). Nevertheless, study [26] developed the Energy-Based Generative Adversarial Network (EBGAN), which is capable of producing low-energy samples by incorporating energy functions into both the generator and discriminator components of the network. A Boundary-Seeking Generative Adversarial Network (BGAN) was developed in Hjelm et al. [27]. This network is used to train the discriminator by assessing the difference between the images that are generated and the images that are being targeted; study [28], on the other hand, presented the Boundary Equilibrium Generative Adversarial Network (BEGAN), which is a framework that combines an equilibrium-based technique to improve the Wasserstein GAN (WGAN) framework.

Several notable applications of GANs have been discovered in existing studies. These applications include object detection [29], handwriting recognition [30], facial age progression [31] as a concept related to face recognition, super-resolution imaging [32], visual saliency prediction [33], and unsupervised domain adaptation [34]. GANs have been used by researchers for data augmentation due to their impressive performance in image synthesis. In the beginning, these techniques were utilized to enhance the overall quality of the photographs, and later on, they were utilized for additional training and the generation of synthetic data. The addition of emotions to neutral faces to increase the number of underrepresented categories has also been performed. Previous studies have explored augmentation techniques and synthetic datasets have used GANs in various domains; for example, Abdulraheem et al. [35], conducted a study in which they leveraged GAN models to develop additional datasets to offer correct results for the automatic recognition of expiration dates in photos. This type of recognition demands a significant number of data for learning purposes. To augment the data, the study installed and evaluated state-of-the-art GAN models, such as WGAN with Gradient Penalty (WGAN-GP), Wasserstein Divergence GAN (WGAN-DIV), Multi-Modal GAN (MMGAN), Non-Saturating GAN (NSGAN), Least Squares GAN (LSGAN), DRAGAN (Deep Regret Analytic GAN), Auxiliary Classifier GAN (ACGAN), Deep Convolutional GAN (DCGAN), Energy-Based GAN (EBGAN), and Variational Autoencoder (VAE). Although not a GAN, VAEs are used for data generation by learning a probabilistic latent space and Boundary Equilibrium GAN (BEGAN). These models were used to augment the existing data. Their method proved that GAN-generated datasets are particularly useful in boosting the overall performance of object identification applications, which is a very promising development. Similarly, in the health domain, the authors of Srivastav et al. [36] conducted a study in which they used deep learning algorithms for the classification of images from chest X-rays to diagnose diseases such as pneumonia. To improve the performance of the model, GANs were trained to augment synthetic images and were then used to oversample the dataset. Image classification was performed using the VGG16 as the base model. The model was able to attain an accuracy of 94.5% when tested on the validation set using the augmented datasets. Another study by the authors of Qin et al. [37] used GAN-generated synthetic datasets alone for the detection of lung diseases. Some of the applied network architectures included state-of-the-art ResNet, DenseNet, EfficientNet, and CNN. The obtained results showed that GAN-generated synthetic datasets achieved improved classification performance. Additionally, for the classification and timely detection of liver lesions, the authors of Frid-Adar et al. [38] also used GAN-based synthetic medical augmentation techniques to increase the performance of a CNN model for up to 85.7% sensitivity and 92.4% specificity. 

The benefits of GAN-based image synthesis and augmentation for brain cancer were highlighted in a review study of data augmentation techniques for brain tumor segmentation by Nalepa et al. [39]. So, the researchers of Safdar et al., and Srinivas et al. [40,41] used these methods to improve brain tumor classification with state-of-the-art models, such as VGG-16 and ResNet-50. Although all of these successes have been achieved in existing studies, there remains a gap in the literature when it comes to the comprehensive comparison of these two approaches (synthetic and augmented datasets) for medical disease prediction, particularly focusing on brain cancer. Some studies have reviewed the use of augmented and synthetic data independently, but they fail to address the critical question of which method is more suitable and effective for medical disease prediction. For instance, the authors of Iglesias., [42] conducted a review of different studies that used augmented and synthetic data, but the study’s scope was too broad and lacked an application-specific focus. On the other hand, the authors of Mirshekarian et al. [43] compared real data with synthetic data for the prediction of blood glucose, but their study did not involve comparing machine learning models with state-of-the-art models, and the aim was different from the premises of this present study. Thus, the need for a study that directly compares augmented and synthetic image datasets using GANs in the context of medical disease prediction remains unaddressed in the existing literature. This research aims to fill this gap and provide insights that have been long-awaited in the medical research community. 

To the best of our knowledge, no previous study has explored this particular aspect in the literature, although some studies, such as Gupta et al., and Mukherkjee et al. [44,45], have employed the use of GANs to increase data size for their classification task using existing machine learning models, without making any clear comparison between synthetic and augmentation techniques to correct the impression that they are the same, making this investigation an original and pioneering effort in the field of disease prediction. By adopting GANs, specifically the sophisticated DCGAN, to generate synthetic datasets of brain and kidney tumors and conduct a direct comparison with the augmented datasets, this research takes a unique approach to address the scarcity of data in medical research and to prevent the risk of an accidental breach and lack of preservation of the privacy of patients represented by datasets. This research also corrects the impression that image augmentation is the same thing as synthetic datasets, which is widely presumed by many researchers. This study also builds a novel C-DCNN model from CNN architectures for the classification of brain tumors and kidney tumors and then conducts a thorough comparison of the performance of our novel C-DCNN model with state-of-the-art models, such as ResNet50, VGG19, InceptionV3, and VGG16, when trained on augmented datasets versus synthetic datasets. The results and insights obtained from this study not only contribute to enhancing predictive accuracy in disease prognosis but also pave the way for further innovations and advancements in medical research using synthetic or augmented data.

## 3. Materials and Methods

**Figure 1:**
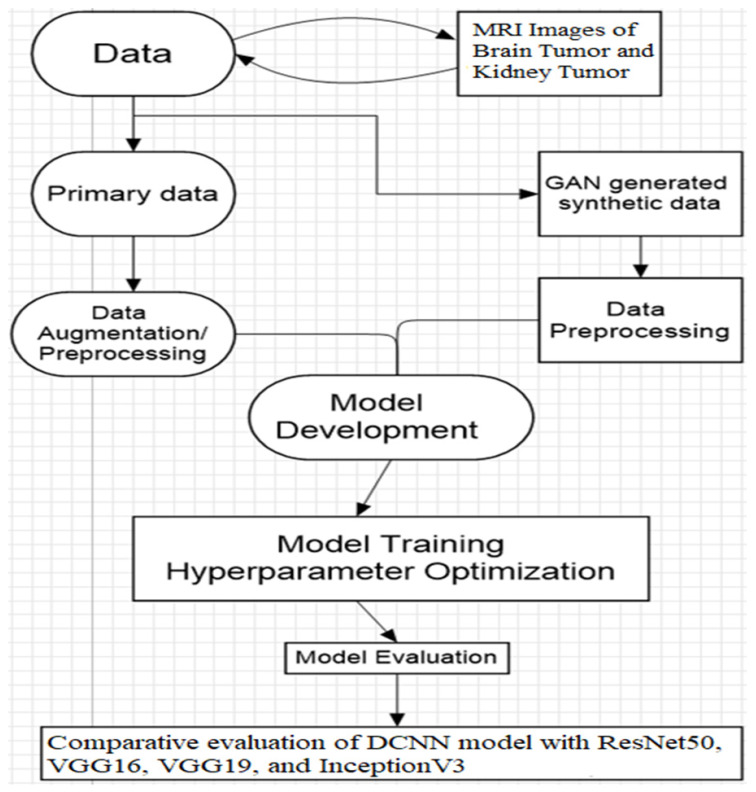
Experimental design and flow of the study.

This section describes the data collecting and analysis processes used to meet the research objectives, together with the experimental design that is depicted in Figure 1. It offers a thorough rundown of all the models, procedures, and strategies used in this study. Model training, testing, optimization, and evaluation were conducted using the Keras package and Python programming language in the Jupyter Notebook environment.

### 3.1. Data Collection

This study relies on data that were obtained from the Cancer Imaging Archive database [46]. This database is available for use in research, and informed consent was obtained to publish the images in an online open-access publication. This dataset includes data from 20 individuals who had primary glioblastoma after diagnosis. Two MRI tests were provided by each patient. All collected images were confirmed to be diseased. The images were in the DICOM format and included binary tumor masks created from T1w images, normalized cerebral blood flow, normalized relative cerebral blood volume, T2w, ADC (apparent diffusion coefficient), FLAIR (fluid-attenuated inversion recovery), and T1w (before and post-contrast agent) images [46]. A dynamic susceptibility contrast (GRE-EPI DSC) imaging post-contrast agent preload was used to create perfusion images. The T1 + C images were co-registered with every series. This dataset provided useful training data for machine learning algorithms in a variety of applications, such as the segmentation and classification of brain tumors. 

Since this was a classification task and all of the acquired image data (brain) were diseased images, we required a different type of disease to train the model. This could enable the model to differentiate between the brain tumor and the other disease. We chose the kidney tumor images in this instance. A supplementary dataset from the segmentation challenge for kidney and kidney tumors in 2019 (KiTS19) training set data [47] was used because the study uses binary classification modeling. This collection used clinical images matched to participants, including scans from 210 University of Minnesota Medical Centre patients who had nephrectomy. The MRI scans, which varied in terms of scanner manufacturers and acquisition techniques, were obtained during standard patient care [47]. The distribution of the data is shown in Table 1.

### 3.2. Image Preprocessing

By eliminating unnecessary variations, image pre-processing increased important characteristics and fine details [48]. Given that all algorithms were susceptible to noise, properly pre-processed images enhanced segmentation and classification tasks [49]. Techniques for pre-processing images can be categorized according to the size of the desired pixel region. These methods work on the sub-images surrounding pixels to reduce noise and distortion and enhance the quality of the image. MRI images can be prevented from being distorted by low image quality, external influences, and a constrained user interface, which can lead to a loss of visual information and processing issues [48]. The regions of interest in the two datasets used in this study were displayed more effectively through image contrast enhancement. DICOM images were initially used to gather MRI images of the kidney and brain tumors. Working directly with DICOM images in CNN frameworks can be difficult because of their non-standard format, lack of software support, and possible compatibility problems. On the other hand, converting DICOM images to the JPG (Joint Photographic Experts Group) format makes managing data easier, minimizes file size, and enhances CNN framework compatibility. The DICOM images were, therefore, converted to the JPG format. Libraries, such as pathlib, shutil, NumPy, os, Pydicom, and PIL, were used for the conversion. The resulting grayscale image was then rescaled, normalized, and saved as a JPG file.

### 3.3. Traditional Data Augmentation Techniques

**Figure 2:**
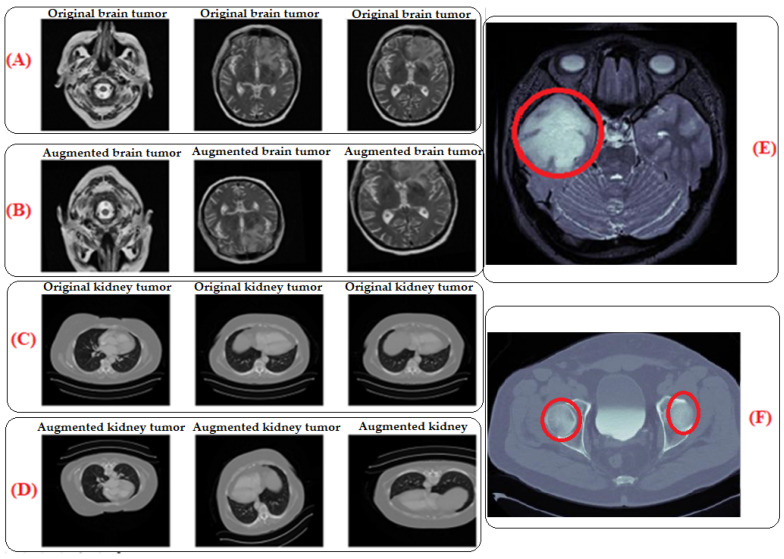
MRI images of original and augmented brain and kidney tumor data. The area circled in red color delineates the tumors. (**A**): Original brain tumor image (This represents the unaltered, initial image used in the training dataset, (**B**): Augmented brain tumor image (This corresponds to the image of the brain image that has undergone traditional data augmentation techniques), (**C**): Original kidney tumor image (This represents the original unaltered MRI scan focused on the kidney, (**D**): Augmented brain tumor image (This corresponds to the image of the kidney tumor, that has undergone traditional data augmentation techniques. (**E**): Magnified view of augmented brain tumor image, (**F**): Magnified view of augmented kidney tumor image.

By subjecting the model to a wider range of variations and scenarios, traditional data augmentation techniques were utilized to expand the size and diversity of our training dataset and improve the model’s robustness [6,50]. This entails using different transformations, such as flipping, shifting, rotating, or zooming, on the current data to produce new training examples that are marginally different from the initial ones, as Table 2 illustrates. By adding more data, the model’s sensitivity to noise or tiny changes in the input data is reduced, improving the generalization performance, as seen in Figure 2. The area circled in the red color shows the tumors. The images in (E) for the brain and (F) for the kidney are the zoomed-in images that show the areas where the tumors are located. (E) represents a zoomed-in view of an augmented brain tumor image and allows for a detailed examination of the specific region where the tumor is located. This zoomed-in view provides insights into how the augmentation process affects the detailed features of the tumor in the image. Similar to part E, (F) shows the augmented kidney tumor image (MRI), which contributes to the diversity of the training dataset for kidney tumor images. (A) The original brain tumor image (MRI) represents the original MRI scan focused on the brain, specifically showcasing a tumor. It is the unaltered, initial image used in the training dataset. (B) The augmented brain tumor image (MRI) corresponds to the image of the brain tumor that underwent traditional data augmentation techniques. These techniques involved applying various transformations like flipping, shifting, rotating, or zooming to create slightly different versions of the original image. Part B displays the augmented version, contributing to an expanded and more diverse training dataset. (C) The original kidney tumor image (MRI) represents the original unaltered MRI scan focused on the kidney, specifically showcasing a tumor. (D) The augmented brain tumor image (MRI) corresponds to the image of the kidney tumor that underwent traditional data augmentation techniques.

To prevent overfitting, which occurs when a model memorizes training data instead of discovering significant patterns, data augmentation is essential. Through data augmentation, randomness and variability are added, making it less likely for the model to overfit and allowing it to develop more reliable and broadly applicable representations [6,51]. We used shear transformations within a maximum shear angle, channel shifting within a specified range, zooming in or out via a specified range, rotation within a certain angle, zero component analysis (ZCA) whitening (disabled in our case), horizontal and vertical flips to mirror images, and width and height shifts to randomly shift images within a fraction of their total width or height. These scales or ranges give the freedom to regulate the degree of transformations used during data augmentation, enabling the customization of the augmentation procedure to the particular needs of the dataset and the deep learning task.

### 3.4. Creating Synthetic Dataset Using Deep Convolutional Generative Adversarial Network (DCGAN)

**Figure 3:**
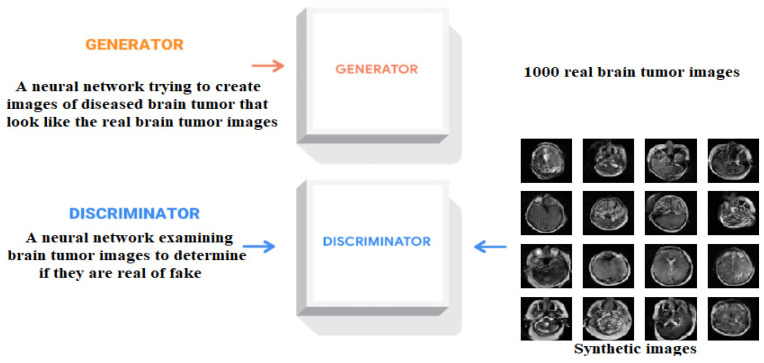
Generator and discriminator phases.

A GAN comprises two distinct phases and two essential components, namely the training phase and the generation phase, with the components being the generator network and the discriminator network [16]. During the training phase, the generator and the discriminator undergo training through an adversarial process. The generator utilizes random input vectors to generate images that possess a realistic appearance, while the discriminator learns to differentiate between real and fake images. During the training process, the generator’s objective is to produce synthetic images that are indistinguishable from real images, while the discriminator’s goal is to accurately categorize both real and synthetic images. The generator gradually improves its ability to generate realistic-looking images, while the discriminator enhances its capacity to distinguish between real and generated images. Equilibrium is achieved in the process when the discriminator becomes unable to differentiate between real and fake images, leading to a competitive drive for both parties to enhance their performance. During the generation phase, once the generator network has been trained to generate artificial data points, random input vectors are selected from the latent space and input into the generator. As shown in Figure 3, the generator converts these arbitrary vectors into artificial data instances, which exhibit the patterns and features acquired during the training process [20]. The synthetic images produced can serve as an independent synthetic dataset or can be merged with preexisting real data to form an augmented dataset. 

**Figure 4:**
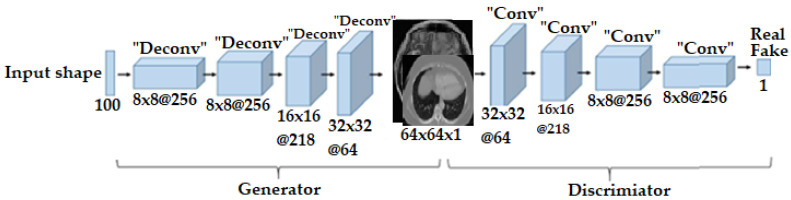
DCGAN architecture of (generator and discriminator).

In this study, DCGAN was employed. DCGAN is a specific type of GAN designed to generate high-quality synthetic images. It was first introduced in [17]. DCGANs employ CNNs as both the discriminator and generator. DCGANs have been extensively utilized and adapted for many imaging production tasks, including object detection, handwriting recognition, facial age progression, and realistic faces and scenes. They have made a substantial contribution to the field of generative models. The DCGAN generator utilizes tf.keras.layers.Conv2DTranspose (upsampling) layers to produce synthetic images from a seed (random noise). Commonly, it consists of layers, such as Conv2DTranspose, Reshape, Dense, etc. For this process, start with a dense layer that takes the seed as its input, then upsample it several times until it reaches the desired image size of 64 × 64 × 1. The generator network (Figure 4) generates a 64 × 64 × 1 brain tumor image from a vector input shape of 100 random integers selected from a uniform distribution. A fully connected layer and four fractionally strided convolutional layers make up the network architecture, which is used to upsample images with a 5 × 5 kernel size. The 64 × 64 × 1 input image is fed into the discriminator CNN architecture, which determines if the lesion is real or fake. This network uses a fully linked layer and four convolution layers, each with a kernel size of 5 × 5. Rather than employing pooling layers to minimize spatial dimensionality, strided convolutions are performed on each convolution layer. The tf.keras.layers.LeakyReLU activation functions for each layer are commonly used, except for the output layer, which uses tanh to ensure that the pixel values are within the range of [−1, 1]. Batch normalization can enhance and accelerate training. The discriminator, as a CNN, is used to distinguish between real images from the dataset and fake images generated by the generator. Usually, it comprises LeakyReLU activation functions and Batch Normalization applied after Conv2D layers. The sigmoid activation of the output layer produces a probability score.

### 3.5. Optimization and Hyperparameter Tuning 

As hyperparameters directly affect the model’s performance and training, they must be optimized, particularly for our DCGAN and the novel model built from CNN architectures. Hyperparameters are configurations or settings that are set by the researcher and not picked up by the model. They have a major impact on the model’s capacity, regularization, and convergence speed [52]. We opted to maintain the hyperparameters of popular models, such as ResNet50, VGG16, VGG19, and InceptionV3, unchanged for the classification task in our study. This choice guarantees the integrity of the knowledge gained by transfer learning, and adjusting these hyperparameters could have a substantial impact on the architecture and functionality of the model. We adjusted the learning rate to be between 0.0002 and 0.0001, the beta 1-exponential decay rate for the first moment estimates in the Adam optimizer to be 0.5, the batch sizes to be 16, 32, 64, and 128 depending on the available computing power, the noise dimension (Latent Dimension) to be 100, and the number of epochs to be between fifty and several thousand depending on the complexity to optimize DCGAN’s hyperparameters. In accordance with complexity, the generator and discriminator architectures were also set. We used the grid-search optimization strategy for our novel C-DCNN model for the classification task. Using a grid-search optimization technique, the optimal configuration for maximizing the model’s performance was found by thoroughly searching through a predetermined set of hyperparameter combinations [53]. This method is important since it allows us to assess the model’s performance in a variety of combinations while methodically exploring the hyperparameter space. By comparing various hyperparameter values, we can determine which configuration is ideal and produces the greatest outcomes for our particular work. The four main hyperparameters that we optimized were the learning rate, batch size, epochs, and optimizer selection. The number of data processed prior to updating the weights in the model was determined by the batch size, which can be anywhere between 10 and 100. The number of epochs, which can be anything between 30 and 100, determines how many times the whole dataset is run through the model while it is being trained. We took into consideration the following seven optimizers: SDG, RMSProp, Adagrad, Adadelta, Adam, Adamax, and Nadam in order to investigate various optimization strategies. Furthermore, we experimented with several learning rates 0.0001, 0.001, 0.01, 0.1, and 0.2 to see how they affected the model’s performance and convergence. Our grid-search optimization revealed that the ideal collection of hyperparameters for our unique C-DCNN model was 32 batches, 50 epochs, an Adam optimizer, and 0.0001 learning rate. These parameters were chosen to optimize the model’s performance on our particular job while preventing problems like slow convergence or overfitting.

### 3.6. Conditional Deep Convolutional Neural Network (C-DCNN) Model

**Figure 5:**
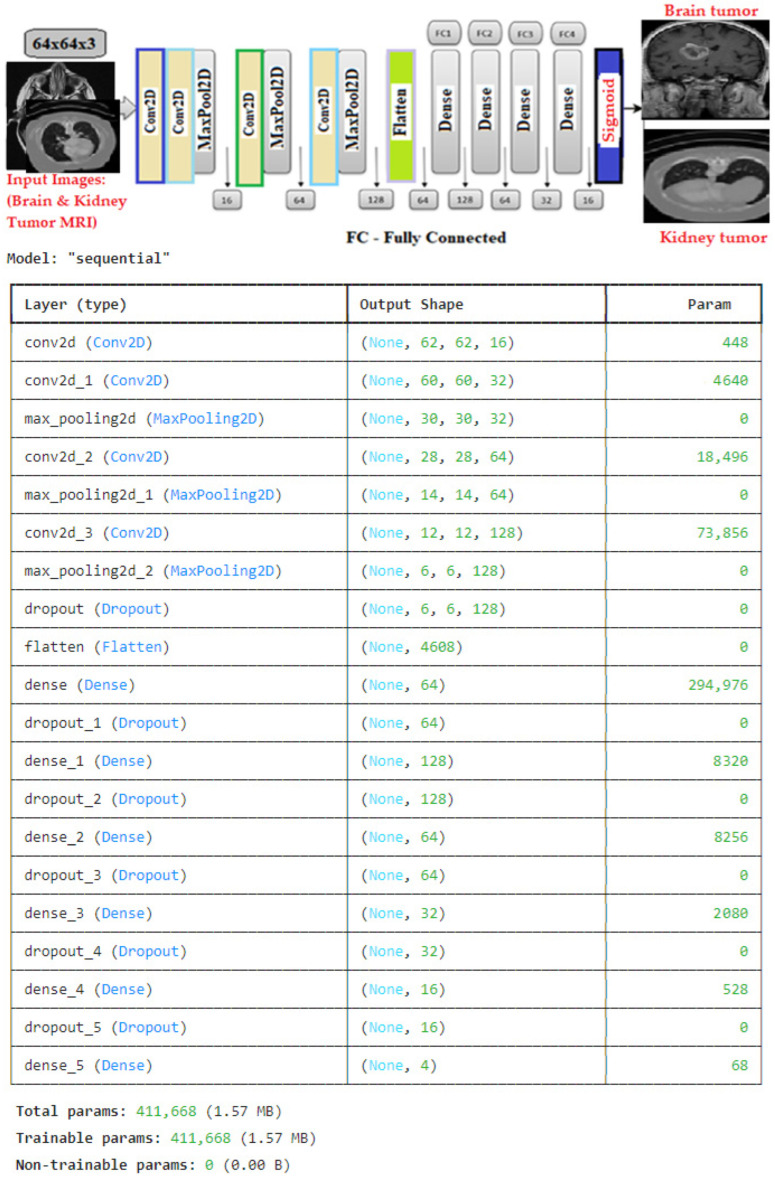
Architecture of the proposed C-DCNN model.

The C-DCNN is an innovative and novel CNN model that utilizes conditional multi-modal contextual fusion to extract distinct features from the dataset. The study utilized the C-DCNN model to showcase the capabilities of a CNN model that was built from scratch, incorporating specific modifications and peculiarity to the data being used to obtain the best possible performance. It aligns with the data utilized and the framework of the problem we aim to address. The C-DCNN model was initially built as a basic CNN model and subsequently enhanced by the addition of layers and the fine-tuning of hyperparameters to maximize its performance. During the process of hyperparameter tuning, we took measures to prevent both overfitting and underfitting in order to achieve sufficient generalization of the unseen data. This may be observed in the model’s performance when compared to the performance of the most advanced pre-trained model assessed in this study. This model is specifically engineered to effectively address both image classification and object recognition tasks. The framework comprises modules specifically designed for extracting features, detecting objects through the region proposal network (RPN), and performing the final classification. We created a CNN model with a well-defined architecture using the classification detection approach. The model includes multiple convolutional and dense layers, as well as dropout for regularization, which helps decide the output information from a single image. The architecture of our CNN comprises a 2D CNN. The network consists of four convolutional layers and three max-pooling layers. The two layers utilized kernel sizes of 3 × 3 and 2 × 2 for pooling. The classification task was accomplished using a sigmoid activation function and a sequence of four fully connected layers. These layers consisted of 128, 64, 32, and 16 neurons, respectively, as depicted in Figure 5. The sigmoid activation function compresses the output of each neuron within the interval of 0 and 1, indicating the likelihood of being classified as part of the positive class. The information pertaining to the training is as follows: the Adam optimizer, which relies on gradients, was used with a batch size of 32. A dropout rate of 25% was applied to the convolutional and fully connected layers, respectively. We employed the binary cross entropy loss function to evaluate the consistency between the anticipated probability and the actual class output, which can only take values of 0 or 1. Ultimately, we proceeded to assemble the model by employing accuracy criteria. The rectified linear activation function (ReLU) was used as the activation function throughout the network, except for the final layer, where the sigmoid activation function was used. Our model is distinguished by its distinctive and innovative approach in multiple dimensions. Firstly, it includes supplementary components, such as batch normalization layers, dropout layers, residual connections, and attention techniques. These changes boost the stability of training, prevent overfitting, improve the flow of gradients, and enable the model to capture long-range dependencies. Furthermore, our model is specifically developed to effectively address image classification tasks. Additionally, it can also be utilized for object recognition tasks, all within a cohesive and integrated framework. This versatile and efficient architecture eliminates the necessity of having distinct models for different activities. Furthermore, the inclusion of an attention mechanism in our model facilitates the capture of interdependencies among spatial locations. This enables the model to concentrate on pertinent regions within the image and comprehend contextual associations. The utilization of this attention mechanism significantly improves performance in activities related to image classification. The combination of convolutional layers and residual connections enhances the model’s ability to effectively learn intricate image features and structures. The convolutional layers collect hierarchical characteristics, while residual connections enhance gradient flow and enable the training of deeper models. Finally, our model is a useful tool for researchers and practitioners due to its adaptability and versatility. 

## 4. Results

This study performed model training and evaluation on augmented datasets and the generated synthetic dataset using the Keras package and Python programming language in the Jupyter Notebook environment. The pre-processed data, totaling 40,000, were split into a training set comprising 80% and a test set comprising 20% for both synthetic and augmentation data. These sets were then fed into the novel C-DCNN model. The model’s performance was assessed by validating it on a randomly selected 20% subset of the training data and later validated on new sets of larger and more diverse datasets of over 18,200 images to confirm our findings. This approach ensured that the model was robust and generalized well to unseen data, highlighting the effectiveness of using both augmented and synthetic data in training. The model was trained for 50 epochs. We implemented the dropout regularization technique following the third max pooling layer and in the thick layers. Dropout regularization is a straightforward and user-friendly technique for regularization. By deactivating certain neurons during the training process, it generates a neural network that is both uncomplicated and effective. A simple neural network leads to lower complexity and, hence, mitigates overfitting. Two callbacks were implemented to enhance the training process, optimize model performance, and prevent overfitting. The first callback, EarlyStopping, monitors the validation loss and terminates the training process prematurely if the loss does not improve for a specified number of epochs. The second callback, ReduceLROnPlateau, reduces the learning rate when the validation loss does not improve for a certain number of epochs, with a patience value of three. 

### 4.1. Validation Metrics

Performance evaluation metrics play a vital role in the development, testing, and deployment of machine learning models, enabling the creation of more precise and efficient AI solutions [54]. They offer a means to objectively assess the model’s accuracy, precision, sensitivity, specificity, and other performance parameters. Performance evaluation metrics allow for the assessment of a model’s performance and provide comparisons across different models. Performance evaluation metrics are crucial for enhancing the transparency and interpretability of machine learning models, which is vital for establishing confidence in these systems. This study assessed the predictive efficacy of the novel C-DCNN model by employing performance evaluation metrics on both synthetic and augmented datasets. The results for both datasets demonstrate that the C-DCNN model achieved exceptional accuracy in detecting and classifying brain tumors and kidney tumors. The augmented dataset yielded a precision of 99% for both types of tumors, while the synthetic dataset achieved a precision of 99% for brain tumors and kidney tumors, as indicated in Table 3. This model’s exceptional precision score suggests its ability to accurately detect brain and kidney tumors in the majority of instances. The recall score of 99% for brain and kidney tumors in both the synthetic and enhanced datasets indicates that this model accurately identified all positive instances. This demonstrates a very responsive model for both artificially generated and augmented datasets with significant therapeutic relevance in precisely identifying tumors in the brain and kidneys in MRI images. The specificity score of 99% for brain tumor and kidney tumors, obtained from both the synthetic and augmented datasets, indicates that the model accurately identified all negative cases. This suggests that the model has a high level of specificity and can effectively determine the absence of a tumor with accuracy. Moreover, an F1 score of 99% achieved by the model for brain tumor and kidney tumors, using both synthetic and augmented datasets, demonstrates its ability to effectively maintain a balance between precision and sensitivity. A high F1 score indicates a favorable equilibrium between precision and recall. Consequently, the model demonstrated an ability to accurately detect instances of brain and kidney tumors while effectively reducing the occurrence of false positive results. The accuracy score of 99% for augmented datasets and 99% for synthetic datasets demonstrates that the model can accurately categorize the generated synthetic and augmented MRI images of brain and kidney tumors and that there is no difference in model performance when augmented or synthetic datasets are used, which further encourages the used of synthetic datasets for classification tasks because of its enormous benefits, especially in increasing primary datasets and circumventing the hurdles of possible breach in data privacy. The C-DCNN model has exceptional efficacy in detecting and precisely categorizing brain and kidney tumors in MRI images. This has profound therapeutic consequences, especially in areas with restricted availability of specialized medical services and resources, as it enables prompt and accurate diagnoses. The C-DCNN model demonstrates the potential to be a powerful tool for detecting and categorizing brain and kidney tumors using CT images. 

### 4.2. Confusion Matrix

**Figure 6:**
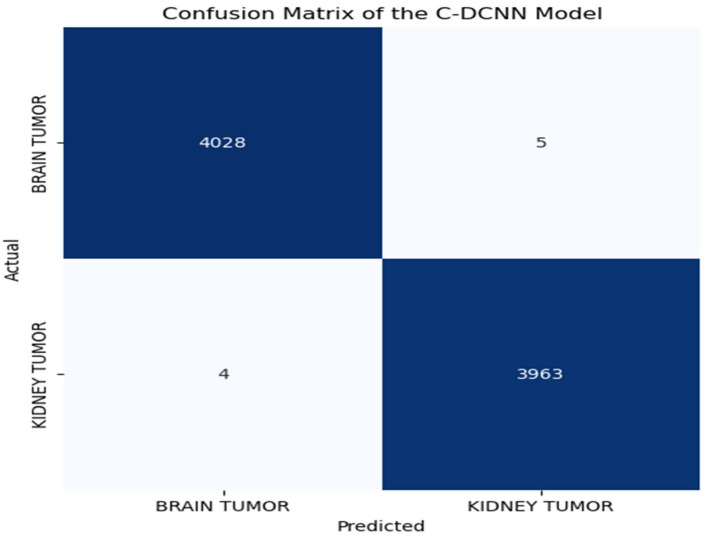
Confusion matrix of the C-DCNN model when fed with synthetic datasets. The blue boxes represent accurately classified tumors, while the white boxes represent misclassified tumors.

**Figure 7:**
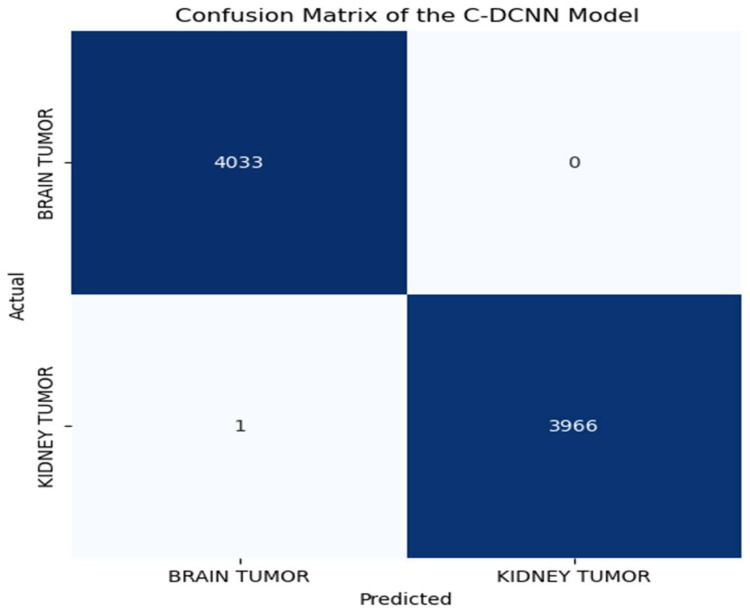
Confusion matrix of the C-DCNN model when fed with augmented datasets. The blue boxes represent accurately classified tumors, while the white boxes represent misclassified tumors.

A confusion matrix is a widely used statistical tool for assessing the performance of models. It aids in assessing the efficacy of our model in accurately categorizing all the images into two distinct classes. The matrix displays the numbers of true positive (TP), true negative (TN), false positive (FP), and false negative (FN) predictions generated by the model. A confusion matrix is crucial for assessing the accuracy and efficacy of models as it offers a concise visual depiction of the model’s performance [55]. Through a thorough analysis of the matrix, one can identify the model’s strengths and weaknesses and implement appropriate enhancements to ensure more precise and dependable diagnoses. The C-DCNN model yielded excellent results, as seen by the confusion matrix. This model accurately identified 4028 MRI images as brain tumors (true positives) while incorrectly classifying 5 images as kidney tumors (false positives), as depicted in Figure 6 for the synthetic datasets. Regarding these augmented datasets, the model accurately identified 4033 MRI images as brain tumors (true positives), while there were no instances where kidney tumors were mistakenly categorized as brain tumors (false positives). This outcome demonstrates that the model exhibits a high level of precision and dependability in the diagnosis of brain tumors. Furthermore, the model accurately classifies 3963 MRI images as kidney tumors (true negatives) and misclassifies 4 as brain tumor images (false negatives) for the synthetic datasets. Similarly, for the augmented dataset, this model correctly classifies 3966 MRI images as kidney tumors (true negatives) but misclassifies 1 as brain tumor images (false negatives), as depicted in Figure 7. The confusion matrix obtained from the C-DCNN model’s detection and classification of MRI images holds great significance in medical diagnosis.

### 4.3. Learning Curve

**Figure 8:**
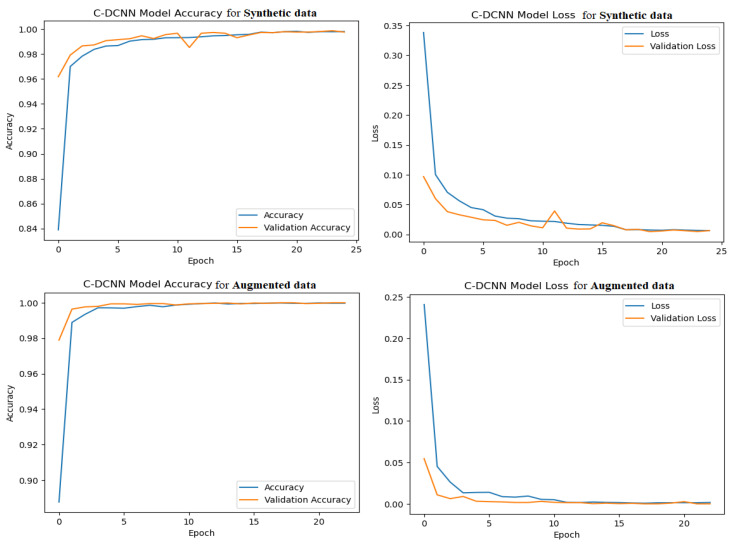
Accuracy and loss of C-DCNN model for augmented and synthetic datasets.

Learning curves are a crucial tool for assessing the effectiveness of these models. It offers insights into the precision and error of a model throughout the training process, enabling the detection of possible performance concerns and providing guidance for enhancing the model. The learning curve of the model accuracy illustrates the progression of this model’s accuracy on both the training and validation datasets as it progresses over time. It can determine whether the model is exhibiting overfitting or underfitting with respect to the training data. An overfitting model exhibits a high level of accuracy when trained on a certain dataset, but its accuracy significantly decreases when tested on a different dataset, suggesting a need for improved generalization to handle new and unfamiliar data. A model that is underfitted can have a poor level of accuracy on both the training and validation data, suggesting that it needs more improvement in order to effectively determine the patterns within the data. By monitoring the learning curve of this model’s accuracy, it becomes feasible to determine the ideal number of epochs for training the model and ensuring that it is neither overfitting nor underfitting. The C-DCNN model demonstrated a robust fit and successfully captured the inherent patterns in the data without experiencing overfitting or underfitting. The progressive enhancement in training accuracy throughout the epochs signified the model’s acquisition and refinement of knowledge. Figure 8 illustrates that the training process began gradually at epoch 0 and consistently improved, resulting in a training accuracy of approximately 99.81% at epochs 25/50 for the synthetic dataset and approximately 99.97% at epochs 23/50 for the augmented datasets. It is crucial to assess the model’s performance on the validation data, which consists of new and unseen data, in order to guarantee that the model is well-balanced with the training data. The training accuracy is equally significant in this evaluation. Like the training accuracy, the validation accuracy initially progresses slowly and encounters a minor inconsistency. Nevertheless, the test’s accuracy gradually rises and reaches a plateau at epoch 25 for synthetic datasets, with an accuracy of 99.89%. Similarly, for augmented datasets, it reaches a plateau at epoch 23 with a test accuracy of 99.99%. This suggests that additional training is unlikely to enhance the model’s performance on fresh data. The model loss learning curve depicts the variation in the loss function of a model during its training across numerous epochs. The training loss diminishes progressively as the model acquires a better match to the data, indicating positive progress. The initial training loss for synthetic datasets was 0.3383 at epoch 1, and it consistently reduced to 0.0065 by epoch 25. Similarly, for augmented datasets, the training loss started at 0.2408 at epoch 1 and steadily decreased to 0.0017 at epoch 23. This suggests that the model enhances its capacity to precisely predict the desired outcome with a reduced margin of error. By contrast, the validation loss exhibited a continuous downward trend from epochs 1 to 17, followed by variability between epochs 18 and 24, and finally, stabilizing at epoch 25 without any consistent drop in the synthetic dataset. The validation loss for the augmented datasets exhibited a steady decline from epochs 1 to 13, followed by fluctuation between epochs 14 and 20, and then stabilizing at epoch 21. From epoch 21 onwards, the validation loss continuously reduced until it reached a plateau at epoch 23. The validation loss quantified the discrepancy between the predicted and actual outputs on an unseen dataset used for evaluating the model’s performance, which was not used for training. Therefore, it calculated the model’s performance using previously unseen data. An optimal fit was attained when the model’s training and validation losses reached a steady state after multiple epochs. Overfitting is indicated by low training loss and high validation loss, but both losses being large may suggest underfitting. Hence, it is imperative to assess the model’s performance and determine the necessary enhancements by scrutinizing the learning curves of model accuracy and model loss.

### 4.4. Receiver Operating Characteristic (ROC) Curve

**Figure 9:**
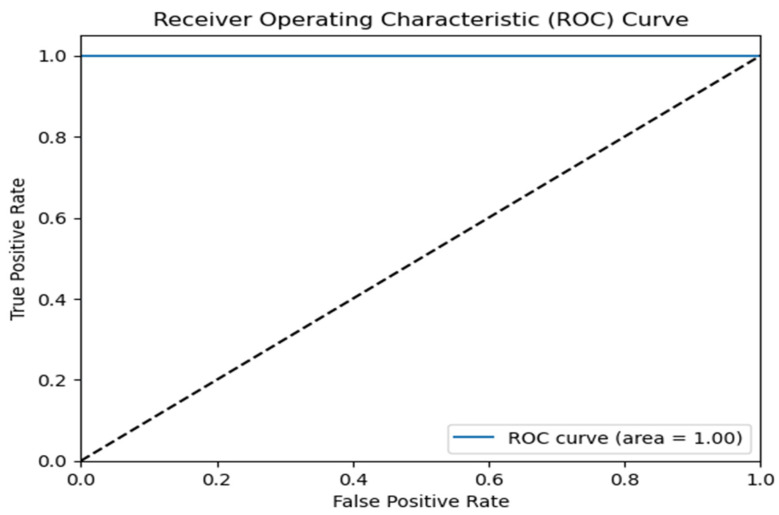
ROC Curve for C-DCNN model for both synthetic and augmented data.

The area under the ROC curve (AUC ROC) is a crucial assessment metric for classification jobs. It offers a visual depiction of the diagnostic accuracy of a classification model. This statistic provides a comprehensive evaluation of a classifier’s performance across all potential classification criteria. The AUC ROC evaluates the sensitivity (true positive rate) and specificity (true negative rate). It offers a singular numerical value that succinctly represents the classifier’s capacity to differentiate brain tumors from kidney tumors. A higher AUC ROC indicates superior model performance. The Receiver Operating Characteristic (ROC) curve is a graphical representation of a binary classification model’s performance as the threshold for discrimination is adjusted. On the other hand, the area under the curve (AUC) is a numerical measure that measures the overall performance of a binary classification model based on its ROC curve. The AUC value measures the overall ability of the model to distinguish between different classes, where a value of 1 indicates perfect classification, and 0.5 suggests random chance [56]. An AUC ROC curve of 1.00 for both synthetic and augmented datasets, as depicted in Figure 9, indicates that the C-DCNN model possesses a strong ability to distinguish between brain tumors and kidney tumors. This indicates that the model has the ability to accurately distinguish between true positive instances (MRI images correctly identified) and false positive cases (MRI images wrongly classified) with a high degree of precision. This model demonstrates outstanding performance in accurately diagnosing brain tumors and kidney tumors, as evidenced by its high AUC value. This indicates that the C-DCNN model has the capacity to offer dependable and precise diagnostic assistance for oncologists.

### 4.5. Comparative Evaluation of C-DCNN Model with State-of-the-Art Advanced Deep Learning Models

By comparing our novel C-DCNN model with other sophisticated deep learning models, like ResNet50, VGG16, VGG19, and InceptionV3, we provide a standard for evaluating its potential to generalize, contribute to progress in the field, and assist in making practical decisions. These models are renowned and extensively utilized transfer learning models in the field of computer vision, exhibiting exceptional performance and making major contributions to tasks such as image recognition and classification [57,58]. Through a comparative analysis of the C-DCNN model with these established models, we can accurately evaluate its performance, competitiveness, and potential superiority. This comparison aims to situate the unique C-DCNN model in relation to current cutting-edge methods and validate its credibility and significance in the field of computer vision. Jupyter Notebooks were utilized for the implementation of all cutting-edge transfer learning models, just like the C-DCNN model. The fine-tuning approach implemented in our innovative C-DCNN model to mitigate the issues of overfitting and underfitting was conducted using all the state-of-the-art models utilized in our comparative evaluation work. 

ResNet50 is a CNN architecture renowned for its use as a profound residual learning methodology. It tackles the issue of diminishing gradients in highly complex networks, enabling the training of exceptionally deep models. It has achieved success in many image classification tasks and is widely recognized for its capability to capture intricate features from CT and MRI images. VGG16 and VGG19 are CNN conceptions that were created by the Visual Geometry Group (VGG) at the University of Oxford. These models exhibit a consistent structure, including numerous layered convolutional and fully linked layers. VGG16 and VGG19 are renowned for their outstanding and unique performances in large-scale image categorization tasks, demonstrating excellent accuracy as a result of their profound and detailed feature extraction capabilities. The InceptionV3, sometimes referred to as GoogleNet, pioneered the idea of inception modules, which effectively capture multi-scale information by utilizing parallel convolutions at various spatial resolutions. This architectural design minimizes computing complexity while still ensuring a high level of accuracy. InceptionV3 has been extensively utilized in many image recognition applications and has exhibited exceptional performance in object detection and localization. 

The comparative evaluation of the novel C-DCNN model with state-of-the-art advanced deep learning models demonstrates differences in performance and offers distinct clinical implications. The C-DCNN model demonstrates superior performance compared to other models, exhibiting enhanced predictive capabilities. The C-DCNN model outperforms VGG16, InceptionV3, VGG19, and ResNet50 in terms of accuracy, precision, sensitivity, specificity, F1 score, AUC, and loss. The model attains 99% accuracy for both synthetic data and augmented datasets, outperforming the other models by a small margin, as demonstrated in Table 4. The C-DCNN model exhibits superior precision, sensitivity, and specificity compared to ResNet50, VGG16, VGG19, and InceptionV3. An F1 score of 99% for both synthetic and augmented data demonstrates an exceptional equilibrium between precision and sensitivity. In addition, the C-DCNN model demonstrates a perfect AUC value of 1.00 for both datasets, demonstrating exceptional discriminative capability. Furthermore, it exhibits a smaller loss value, suggesting superior optimization and a reduced number of errors. The enhanced precision of the C-DCNN model has notable clinical importance. The C-DCNN model achieves a perfect classification accuracy of 99% for identifying brain and kidney tumors in MRI images, ensuring dependable and accurate outcomes. Oncologists and healthcare professionals engaged in the diagnosis and treatment of these tumors can considerably benefit from this exceptional level of accuracy. It decreases the likelihood of misdiagnosis, facilitating prompt identification and suitable response, hence enhancing patient outcomes. The C-DCNN model surpasses previous models in multiple criteria, making it a more reliable and precise tool for aiding oncologists in crucial decision making. 

The exceptional efficacy of the novel C-DCNN model can be ascribed to various variables, including its distinctive architectural configuration, efficient training methodology, and enhanced capacity to acquire and depict the pertinent characteristics in brain and kidney tumors. The inclusion of particular design elements, such as the Mixed-Scale Dense Convolution Layer, Self-Attention Mechanism, Hierarchical Feature Fusion, and Attention-Based Contextual Information, allowed the C-DCNN model to more efficiently capture and extract pertinent features for brain tumor classification. The C-DCNN model was developed utilizing an optimized configuration and successful training methodologies, including the meticulous selection of hyperparameters, such as the learning rate, batch size, and regularization approaches. These combinations expedite the process of convergence and enhance the model’s ability to discover a more optimal solution. Although ResNet50 is a complex model renowned for its deep architecture and skip connections, it demonstrated the lowest performance when compared to other models. The performance metrics indicate that ResNet50 achieved an accuracy of 93.67% for synthetic data and 92.59% for augmented data, a precision of 92.6%, and an F1 score of 92.6% for augmented datasets. However, these results reveal that ResNet50 had a relatively larger number of false positives and false negatives, leading to less-than-optimum predictions. This outcome may arise because of the distinctive attribute of the utilized data and the intricate structure of the ResNet50 model. The InceptionV3, also known as the GoogleNet model, exhibited somewhat inferior performance compared to VGG19, which is an enhanced version of the VGG16 model. This is evident in its lower sensitivity, specificity, F1 score, true positive rate (TP), area under the curve (AUC), and accuracy. This discrepancy can potentially be ascribed to reasons such as the heightened intricacy of the InceptionV3 and VGG19 models, resulting in an inadequate depiction of the particular features pertinent to the categorization tasks. 

### 4.6. Expert Assessment of DCGAN-Generated Synthetic Data

To evaluate the synthesized data of brain and kidney tumors, we tasked two experienced radiologists with categorizing real, augmented, and synthetic images for both datasets. This experiment aimed to determine whether radiologists could distinguish between the real images and synthetic images generated by the DCGAN. Consistent classification outcomes would indicate the utility of the artificial data for training machine learning models.

Evaluation Process:
Image Presentation: The experts were shown a randomized mix of real primary images, augmented images, and synthetic images. Each set included the following:

- Original brain tumor images (MRI);
- Augmented brain tumor images (MRI);
- Synthetic brain tumor images generated by the DCGAN;
- Original kidney tumor images (MRI);
- Augmented kidney tumor images (MRI);
- Synthetic kidney tumor images generated by the DCGAN.

Task: The radiologists were asked to classify each image as either real (original/augmented) or synthetic.
Scoring: The accuracy of their classifications was recorded, with a focus on the difference in performance between real and synthetic images.
Results:

- Expert 1: accurately recognized both real and synthetic brain and kidney tumors with a 20% success rate.
- Expert 2: accurately classified the real, augmented, and synthetic brain and kidney images with a 25% success rate for each category.

Both experts achieved comparable classification performances for real, augmented, and synthetic images. This indicates that the synthetic images generated using the DCGAN have significant similarity to real and augmented images, thus validating their potential utility for training machine learning models.

**Figure 10:**
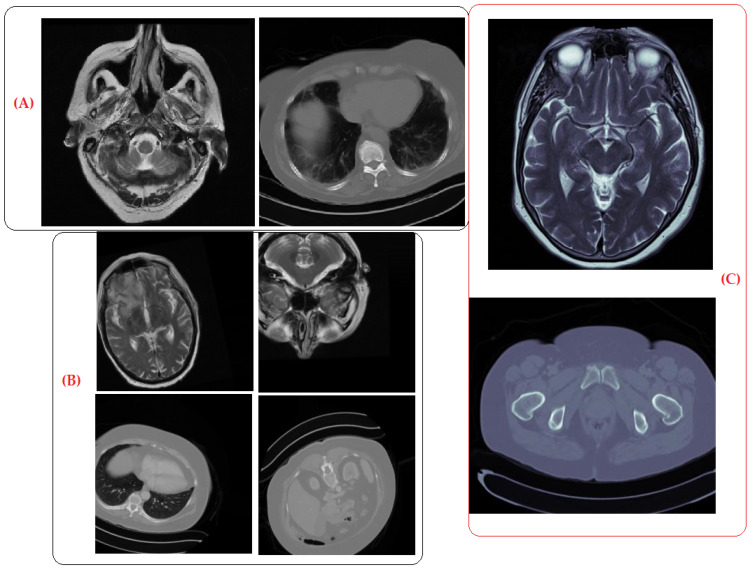
(**A**) Original, (**B)** augmented, and (**C**) synthetic images generated by DCGAN.

*Sample Images:* To provide further clarity on the quality of the synthetic images, we include sample images in Figure 10.

The comparable performance of the radiologists in categorizing real, augmented, and generated images confirmed the efficacy of the DCGAN in producing realistic synthetic images. The DCGAN not only generated new images but also provided notable benefits, such as the ability to generate synthetic images with diverse variations, enhance image qualities, and streamline the training process by eliminating artifacts. These images can be confidently used for training machine learning models, enhancing the diversity and robustness of the datasets to enhance the accuracy of model performance.

### 4.7. Data Quality Assessment Using MSE

To evaluate the quality of synthetic images generated by the DCGAN, we further calculated the Mean Squared Error (MSE) between the original and synthetic images. MSE measures the average squared difference between the original and synthetic images, providing a quantitative assessment of image quality and thereby providing insights into the effectiveness of the DCGAN in generating high-quality synthetic data.

To calculate the MSE between the original and synthetic images, we followed these steps:

Step 1: Preprocess the images to ensure they are in the same format and size.
Step 2: Compute the pixel-wise differences between the original and synthetic images.
Step 3: Square the differences.
Step 4: Compute the mean of the squared differences.

The formula for MSE is as follows:

$$MSE=\frac{1}{N}\;\sum_{i=1}^{N}\;(Ii-Si{)}^{2}$$

where Ii is the pixel value of the original image, Si is the pixel value of the synthetic image, and *n* is the total number of pixels when the implementation of the MSE was computed in the Python environment using NumPy version: 1.24.3.

Results:

The MSE was computed for a representative sample of original and synthetic images from the brain tumor and kidney tumor datasets. With a pixel value in the range [0, 255], the results showed an average MSE of 0.166, indicating that the synthetic images closely resembled the original images. These findings support the effectiveness of the DCGAN in generating high-quality synthetic data that can be used for model training and evaluation.

## 5. Conclusions

In conclusion, this study introduces a technique that emphasizes the medical importance of deep learning models, particularly the novel C-DCNN model, in the detection and diagnosis of brain tumors when fed with MRI images generated by GANs, specifically the sophisticated DCGAN and images augmented through traditional techniques, such as manipulating the training set by changing its geometric and color space properties (such as rotation, scaling, cropping, brightness, zooming, and contrast) to enhance classification accuracy, and comparing the performances of state-of-the-art deep learning models, such as ResNet50, VGG16, VGG19, and InceptionV3 when trained on augmented and synthetic datasets. We applied this methodology to brain and kidney tumor images. The C-DCNN model, built entirely from scratch using CNN architectures, demonstrated exceptional accuracy, precision, sensitivity, specificity, and F1 score, underscoring its potential as an efficient tool for swift and precise diagnosis. The novel C-DCNN model demonstrated its performance efficiency by effectively classifying between brain and kidney tumors when trained with augmented and synthetic datasets.

The primary discovery of this study is that synthetic datasets created using GANs can achieve comparable performance to augmented datasets when utilized for training deep learning models. This is consistent with studies such as [23,59,60] that have recorded improved accuracy utilizing synthetic datasets generated using GANs. This demonstrates the considerable potential of synthetic datasets to be widely used as an established augmentation technique for deep learning in industrial applications. These applications typically feature small-sized datasets and offer many advantages, such as privacy preservation. The proposed DCGAN (Deep Convolutional Generative Adversarial Network) for generating synthetic data provides a considerable advantage by reducing the time and opportunity costs associated with collecting real-world data. The results of this work enhance the continuous progress in AI-driven cancer diagnosis and highlight the capability of deep learning models to enhance diagnostic precision and patient care, even when trained on synthetic datasets.

Despite the promising findings, several limitations warrant our acknowledgment. Firstly, our study primarily compared the novel C-DCNN model with state-of-the-art models, such as ResNet50, VGG16, VGG19, and InceptionV3. While these models are well-regarded in the literature, there may be other models with superior performance that we did not consider. Future studies should include a broader range of sophisticated models to ensure a comprehensive comparison with our novel C-DCNN model. Secondly, our study utilized the DCGAN to generate synthetic images. While the DCGAN is effective, there are more advanced models available that might produce higher-quality synthetic images. Future research should explore and test other GAN architectures to determine if they offer improved image-generation capabilities. Future research could incorporate additional clinical factors and expert insights to refine the evaluation and selection criteria for these models. This would provide a more holistic approach to the model assessment and potentially improve performance. To enhance the applicability and generalizability of our findings, future studies should expand the scope of our analysis to include a broader range of imaging modalities and cancer types. This can enable the model to be applicable in diverse clinical scenarios and improve its robustness. Finally, our study focuses on binary classification. Extending the research to multi-class classification problems is an important area for future work. This would allow the model to handle more complex scenarios and provide more detailed diagnostic information.

Incorporating synthetic data and the C-DCNN model into radiological workflows holds significant promise for improving diagnostic accuracy, enhancing training, and supporting clinical decision making. However, addressing the outlined limitations is essential for successful integration. Future research should focus on validating these technologies, gaining acceptance among medical professionals, ensuring technical compatibility, maintaining data privacy, and establishing frameworks for continuous learning.

Our future plans involve expanding our work to include additional medical fields that can benefit from synthetic data produced using GANs. This expansion can enhance training efforts and lead to improved classification outcomes across various medical disciplines. By leveraging synthetic data, we aim to address data scarcity issues and improve the robustness and accuracy of diagnostic models.

## Figures and Tables

**Table 1:** Distribution of data.

References	Class Data	Class Distribution	After Augmentation	Synthetic Data Using DCGAN	Training/Testing Dataset	Label
(Clark et al., 2013) [46]	Brain Tumor	1000	20,000	20,000	80/20	1
(Heller et al., 2019) [47]	Kidney Tumor	1000	20,000	20,000	80/20	0

**Table 2:** Data augmentation techniques and their range.

	Techniques	Range/Scale
0	Horizontal flip	True
1	Vertical flip	True
2	Width shift range	0.3
3	Height shift range	0.3
4	Shear range	0.2
5	Zoom range	0.2
6	Rotation range	0.2
7	ZCA whitening	False
8	Channel shift range	0.2

**Table 3:** Validation metrics of the novel C-DCNN model.

Type of Dataset	Class Data	Precision %	Sensitivity %	Specificity %	F1-Score %	Accuracy %
Synthetic Data	Brain Tumor	99	99	99	99	99
Augmented Data	Kidney Tumor	99	99	99	99	99

**Table 4:** Comparative evaluation of C-DCNN model with state-of-the-art advanced deep learning models.

Type of Dataset	Models	Precision %	Sensitivity %	Specificity %	F1-Score %	TP	FP	TN	FN	AUC	Loss	Accuracy %
Synthetic Data	C-DCNN	99	99	99	99	4028	5	3963	4	1.00	0.002	99
	VGG19	97.8	97.8	97.8	97.8	4027	6	3954	13	0.99	0.005	97.76
	VGG16	97.9	97.9	97.9	97.9	4024	9	3962	5	0.99	0.004	97.83
	ResNet50	93.6	93.6	93.6	93.6	3950	83	3704	263	0.72	0.111	93.67
	InceptionV3	97.7	97.7	97.7	97.7	4017	16	3956	11	0.99	0.011	97.66
Augmented Data	C-DCNN	99	99	99	99	4033	0	3966	1	1.00	0.001	99
	VGG19	97.9	97.9	97.9	97.9	4032	1	3964	3	0.99	0.003	97.95
	VGG16	97.9	97.9	97.9	97.9	4032	1	3965	2	1.00	0.001	97.96
	ResNet50	92.6	92.6	92.6	92.6	3919	114	3648	319	0.99	0.156	92.59
	InceptionV3	97.8	97.8	97.8	97.8	4021	12	3963	4	0.99	0.007	97.80

## Data Availability

Data can be found on the cancer imaging archive webpage https://wiki.cancerimagingarchive.net/display/Public/Brain-Tumor-Progression#33948119f2aee006de854c85aea7ef6b9bf5293a (accessed on 5 December 2023) and https://wiki.cancerimagingarchive.net/pages/viewpage.action?pageId=61081171 (accessed on 5 December 2023). * Access permission is needed for usage.
